# Clinical phenotypes and genetic mutation analysis of 45 neonatal-onset methylmalonic acidemia

**DOI:** 10.3389/fped.2026.1708194

**Published:** 2026-06-05

**Authors:** Qun Xu, Lili Kang, Huiting Yv, Yunxia Li, Chen Liu, XiaoYing Li

**Affiliations:** Department of Neonatology, Children’s Hospital Affiliated to Shandong University, Jinan, Shandong, China

**Keywords:** clinical characteristics, gene mutation, methylmalonic acidemia, MMACHC gene, MMUT gene, neonatal onset

## Abstract

**Background:**

Methylmalonic acidemia (MMA), the most prevalent organic acidemia in China, is an autosomal recessive disorder. Neonatal-onset MMA often presents with non-specific manifestation, often causing diagnostic delays.

**Methods:**

A retrospective analysis was conducted on the clinical data, laboratory findings, and genetic information of 45 neonatal-onset MMA patients admitted to the Children's Hospital Affiliated to Shandong University from October 2016 to June 2024.

**Results:**

1) Classification: 32 combined MMA, 13 isolated MMA. Median onset: 11 days. Common manifestations: feeding difficulties (60.0%), failure to thrive (57.8%), jaundice (46.7%), respiratory distress (40.0%), impaired consciousness (28.9%). Key labs: hyperammonemia (40.0%), macrocytic anemia (33.3%), granulocytopenia (31.1%). Hydrocephalus occurred in 3/31, abnormal EEGs in 15/19. Elevated C3, C3/C0, methylmalonic acid, and methylcitric acid were diagnostic. 2) Combined MMA had significantly lower methionine, blood ammonia, and C3/C0 vs. isolated MMA. Isolated MMA showed lower leukocyte counts (*p* < 0.05). 3) Whole-exome sequencing: 31/32 (96.8%) had *MMACHC* mutations; c.609G>A recurrent (12/31). One had ABCD4 mutation. Isolated MMA: All 13 had MMUT mutations; c.729_730insTT common (46.2%). Missense mutations predominated.

**Conclusion:**

Neonatal-onset MMA presents with non-specific clinical phenotypes. Therefore, unexplained feeding difficulties, neutropenia, hyperammonemia, or seizures warrant prompt homocysteine, tandem MS, and urinary organic acid screening. Isolated MMA shows earlier free carnitine decline and higher ammonia. *MMACHC* c.609G>A and MMUT c.729_730insTT are recurrent in combined and isolated subtypes, respectively.

Methylmalonic acidemia (MMA) is a congenital disorder of organic acid metabolism, primarily caused by a deficiency in methylmalonyl-CoA mutase (MCM) or defects in the intracellular metabolism of cobalamin (vitamin B12). These abnormalities disrupt the conversion of methylmalonyl-CoA, a key intermediate in the catabolism of isoleucine, valine, methionine, threonine, cholesterol, and odd-chain fatty acids, into succinyl-CoA. As a result, alternative metabolic pathways are upregulated, leading to the accumulation of toxic metabolites such as methylmalonic acid, 3-hydroxypropionic acid, and methylcitric acid. Based on biochemical profiles, MMA can be classified into two major types: isolated MMA and combined MMA. To date, more than ten disease subtypes related to MMA have been identified (15), with the majority exhibiting autosomal recessive inheritance. The disease can manifest at any age and presents with a wide range of clinical symptoms (1). Without timely diagnosis and intervention, MMA can lead to multi-organ damage and may be life-threatening. In neonates, MMA often may presents insidiously, with nonspecific clinical signs that Diagnostically challenging. Common early symptoms—including feeding difficulties, vomiting, lethargy, and seizures—closely resemble those seen in neonatal sepsis, hypoglycemia, or hypoxic-ischemic encephalopathy (HIE), contributing to frequent misdiagnosis or delayed diagnosis and treatment, which may adversely affect outcomes. Early diagnosis and prompt intervention are therefore essential for improving prognosis.

To enhance clinical understanding of neonatal-onset MMA, this study retrospectively analyzes the clinical features, routine laboratory findings, mass spectrometry results, and genetic testing data of 45 neonates diagnosed with MMA and admitted to our hospital between October 2016 and June 2024. Our aim is to elucidate the clinical presentation, biochemical and mass spectrometry characteristics, and genetic mutation profiles of neonatal-onset MMA, thereby increasing clinician awareness and to support earlier recognition and management.

## 1 Study subjects

The study enrolled neonates diagnosed with methylmalonic acidemia (MMA) who were admitted to the Department of Neonatology at the Children's Hospital Affiliated to Shandong University between October 2016 and June 2024. All patients were onset in neonatal period. Genetic diagnosis was confirmed following informed consent from the parents or guardians and completion of a signed consent form for genetic testing. The study protocol was approved by the Institutional Review Board of the hospital (approval number: SDFE-IRB-P-2023034) and was supported by the Jinan Municipal Health Commission Science and Technology Program in 2023 (grant number: 2023-2-142).

## 2 Methods

### 2.1 Selection and exclusion criteria of research subjects

Patient selection criteria: All 45 patients were genetically confirmed cases, with complete acylcarnitine screening and urine organic acid analysis, meeting C3 > 5 µmol/L and urinary MMA >4; among them, six cases were newborn screening positive with no obvious clinical symptoms; cases with complex congenital heart disease, other organ malformations, chromosomal abnormalities, or other definite genetic metabolic diseases were excluded. 2.2 Biochemical Blood Tests, Acylcarnitine Profile Screenin, Urinary Organic Acid Measurement, and Other Auxiliary Tests.

Blood gas analysis, transaminases (ALT/AST), lactate levels, plasma ammonia, homocysteine, cardiac enzyme profile, and blood glucose levels were measured as part of routine biochemical testing. For neonates suspected of having organic acidurias, peripheral blood samples were collected and spotted onto specialized blood collection filter paper, which was air-dried at room temperature before analysis. Blood amino acids and acylcarnitine levels were measured using tandem mass spectrometry (MS/MS).For patients with positive acylcarnitine screening(C3 > 5umol/L), a urine sample (at least 5 mL) was collected for urinary organic acid analysis using gas chromatography-mass spectrometry (GC-MS).

### 2.3 Pathogenic analysis of gene mutation

After obtaining informed consent from the guardians and approval from the hospital's ethics committee, 2 mL venous blood samples were collected from both the child and parents.Whole-exome next-generation sequencing (WES) was performed as the high-throughput sequencing method to comprehensively detect the exonic regions of all protein-coding genes, and any disease-related mutations were identified. These mutations were then validated in the parents using Sanger sequencing, the gold standard for genetic variation verification.

### 2.4 Treatment and follow-up

For confirmed cases, treatment included vitamin B12 supplementation (1–2.5 mg/d, im/iv), L-carnitine(100–200 mg/kg d, oral), and in the case of combined MMA, betaine(1 g/d) to lower homocysteine levels. Isolated MMA cases were treated with dietary control (MMA special formula milk and ordinary stage 1 formula milk in a ratio of 3–4:1). Follow-up visits were scheduled 1–2 months post-discharge, and later every 3–6 months once the condition was stable. Follow-up assessments included growth and cognitive development evaluations, homocysteine levels, plasma ammonia, liver function tests, acylcarnitine profile, and urinary organic acid measurement.

### 2.5 Statistical analysis

**Figure 1:**
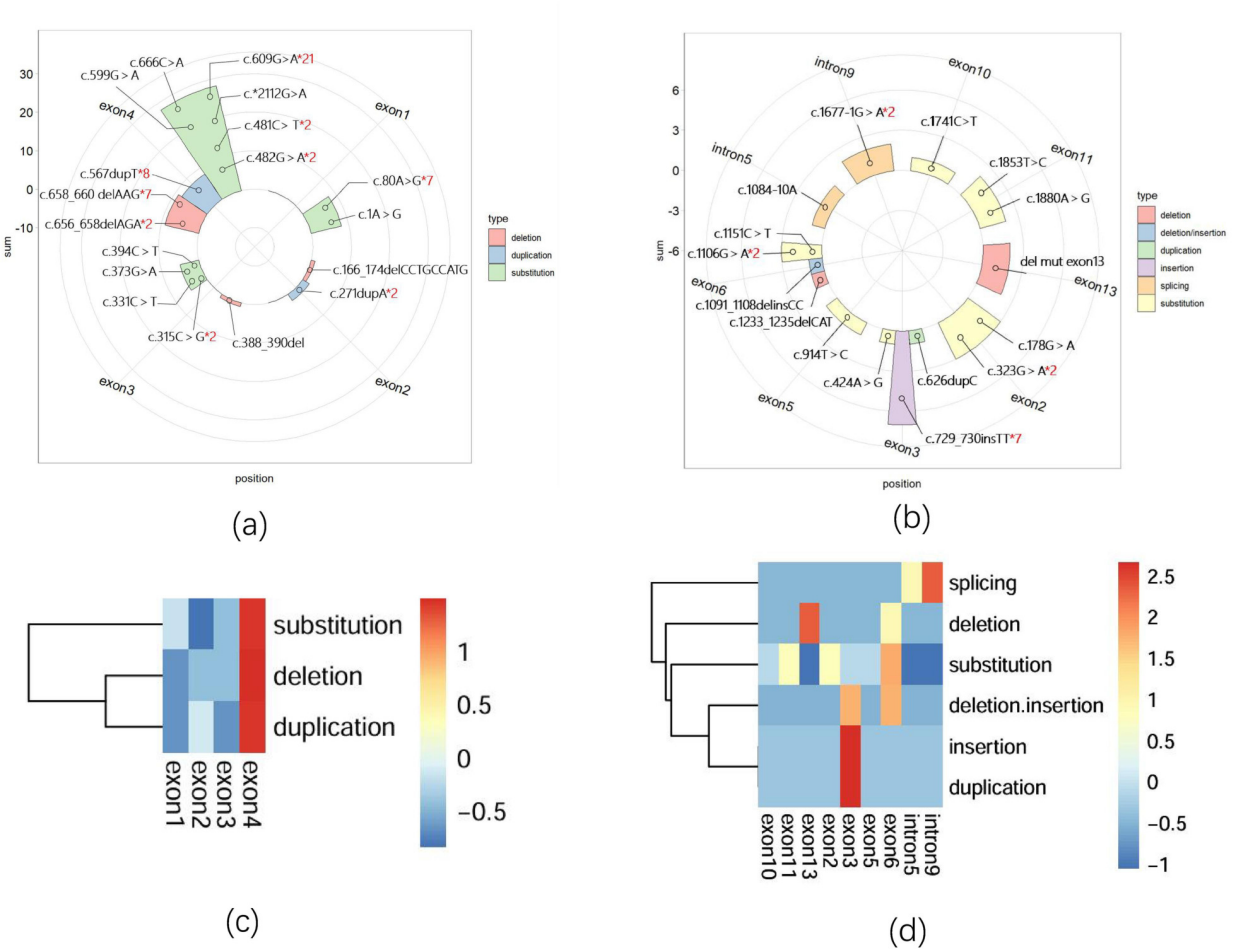
**(a)** *MMACHC* gene mutation sites identified in patients with combined methylmalonic acidemia in our center; **(b)** *MMUT* gene mutation sites identified in patients with isolated methylmalonic acidemia in our center; **(c)** distribution of *MMACHC* gene mutation types in patients with combined methylmalonic acidemia in our center; **(d)** distribution of *MMUT* gene mutation types in patients with isolated methylmalonic acidemia in our center.

MMA patients were divided into two groups: combined MMA and isolated MMA, based on the presence or absence of hyperhomocysteinemia. Clinical features, laboratory test results, and gene phenotypes were compared between the two groups. Data analysis was performed using R version 4.3.1. Normally distributed continuous variables were expressed as mean ± standard deviation (SD) and compared between groups using t-tests. Non-normally distributed continuous variables were expressed as median and interquartile range (IQR), with comparisons made using the Wilcoxon rank-sum test. Categorical data were described using frequencies and percentages (*n*%), and group comparisons were conducted using the chi-square test or Fisher's exact test. A *p*-value of <0.05 was considered statistically significant (Figure 1).

Gene mutation data for combined MMA and isolated MMA were analyzed, and circular bar plots were created to show the exonic locations of *MMACHC* and *MMUT* gene mutations, with specific mutation sites annotated. Heatmaps were generated to visualize the distribution of nucleotide mutation types in the *MMACHC* and *MMUT* genes. All visualizations were performed using R Studio (version R 4.3.2).

## 3 Results

### 3.1 General characteristics

Among the 45 pediatric patients included in this study, 26 were male and 19 were female. A total of 43 cases were full-term infants, while 2 were preterm, with gestational ages of 33 weeks and 36^+6^ weeks, respectively. The mean birth weight was 2,977 ± 402 grams. Of these 45 patients, 18 had a positive family history. Specifically, 17 cases had a maternal history of unexplained miscarriage, and 1 case involved a sibling who died of unknown causes shortly after birth.

According to the presence or absence of concomitant hyperhomocysteinemia, patients were categorized into two subtypes: combined MMA (methylmalonic acidemia with hyperhomocysteinemia) and isolated MMA. Among the 45 cases, 32 (71.1%) were classified as combined MMA, while 13 (28.9%) were isolated MMA. No significant differences were observed between the two groups in terms of sex distribution, gestational age, birth weight, maternal history of miscarriage, or sibling death. Detailed data are presented in Table 1.

**Table 1:** General characteristics of combined type and isolated type MMA groups.

Variable	Level	Total (*n* = 45)	Combined type (*n* = 32)	Isolated type (*n* = 13)	*p* value
Sex	Male	26 (57.78)	19 (59.38)	7 (53.85)	0.9941
	Female	19 (42.22)	13 (40.62)	6 (46.15)	
Fetal age	Preterm	2 (4.44)	1 (3.12)	1 (7.69)	0.4990
	Full-term	43 (95.56)	31 (96.88)	12 (92.31)	
Birth weight (g)	Mean ± SD	2,977 ± 402	2,987 ± 334	2,952 ± 552	0.7988
Maternal history of abortion	−	28 (62.22)	21 (65.62)	7 (53.85)	0.5114
	+	17 (37.78)	11 (34.38)	6 (46.15)	
Sibling death	−	44 (97.78)	31 (96.88)	13 (100.00)	1.0000
	+	1 (2.22)	1 (3.12)	0 (0.00)	
Age at onset (d)	Median	11 [4, 18]	15 [4.00, 19.25]	5 [3, 8]	0.0360

### 3.2 Clinical phenotypic features

#### 3.2.1 Onset age

The median age at onset among the 45 patients was 11 days. A total of 19 cases (42.2%) presented within the first 7 days of life, 7 cases (15.6%) between 8 and 14 days, and 19 cases (42.2%) between 15 and 28 days. In the combined MMA group, the median onset age was 15 days (4, 19.25), while in the isolated MMA group, it was 5 days (3, 8). Compared to the combined MMA group, the isolated MMA group had an earlier onset, and the difference was statistically significant (*p* *<* 0.05) (Table 1).

#### 3.2.2 Clinical phenotypic characteristics

Among the 45 pediatric patients, 27 (60.0%) presented with feeding difficulties, 26 (57.8%) with poor weight gain, 21 (46.7%) with jaundice, 18 (40.0%) with dyspnea, 13 (28.9%) with consciousness disorder, 9 (20.0%) with seizures, 11 (24.4%) with limb tremors, and 4 (8.8%) with recurrent vomiting. Compared with patients with combined type MMA, those with isolated type MMA had a significantly higher incidence of dyspnea (*p* < 0.05). No statistically significant differences were observed in other clinical manifestations between the two groups (Table 2).

**Table 2:** Comparison of clinical symptoms between combined type and isolated type MMA groups.

Variable	Level	Total (*n* = 45)	Combined Type (*n* = 32)	Isolated Type (*n* = 13)	*p* value
Feeding difficulty	−	18 (40.00)	14 (43.75)	4 (30.77)	0.6384
	+	27 (60.00)	18 (56.25)	9 (69.23)	
Poor weight gain	−	19 (42.22)	15 (46.88)	4 (30.77)	0.5102
	+	26 (57.78)	17 (53.12)	9 (69.23)	
Jaundice	−	24 (53.33)	19 (59.38)	5 (38.46)	0.3447
	+	21 (46.67)	13 (40.62)	8 (61.54)	
Dyspnea	−	27 (60.00)	24 (75.00)	3 (23.08)	0.0039
	+	18 (40.00)	8 (25.00)	10 (76.92)	
Consciousness disorder	−	32 (71.11)	25 (78.12)	7 (53.85)	0.1493
	+	13 (28.89)	7 (21.88)	6 (46.15)	
Seizures	−	36 (80.00)	26 (81.25)	10 (76.92)	0.7037
	+	9 (20.00)	6 (18.75)	3 (23.08)	
Limb tremors	−	34 (75.56)	24 (75.00)	10 (76.92)	1.0000
	+	11 (24.44)	8 (25.00)	3 (23.08)	
Recurrent vomiting	−	41 (91.11)	29 (90.62)	12 (92.31)	1.0000
	+	4 (8.89)	3 (9.38)	1 (7.69)	
Outcome	Improved	32 (71.11)	26 (81.25)	6 (46.15)	0.0300
	Deceased	13 (28.89)	6 (18.75)	7 (53.85)	

### 3.3 Laboratory and imaging findings

#### 3.3.1 Routine laboratory tests

Among the 45 pediatric patients, hyperammonemia was observed in 18 cases (40.0%), metabolic acidosis in 17 cases (37.8%), macrocytic anemia in 15 cases (33.3%), neutropenia or agranulocytosis in 14 cases (31.1%), and elevated transaminases in 6 cases (13.3%). When comparing the combined type and isolated type MMA groups, hyperammonemia was significantly more common in patients with isolated type MMA (*p* < 0.05). The median plasma ammonia level in the isolated type group was 337.5 µmol/L, compared to 55.5 µmol/L in the combined type group, and this difference was statistically significant (*p* *<* 0.05). All patients with isolated type MMA developed metabolic acidosis, which was significantly more frequent than in the combined type group (*p* < 0.05). There were no significant differences between the two groups in terms of neutropenia/agranulocytosis, macrocytic anemia, abnormal liver function, or mean corpuscular volume (MCV) (Table 3).

**Table 3:** Comparison of laboratory findings between combined type and isolated type MMA groups.

Variable	Level	Total (*n* = 45)	Combined type (*n* = 32)	Isolated type (*n* = 13)	*p* value
Hyperammonemia	−	25 (56.82)	22 (70.97)	3 (23.08)	0.0069
	+	18 (40.91)	8 (25.81)	10 (76.92)	
Metabolic acidosis	−	27 (61.36)	27 (87.10)	0 (0.00)	0.0000
	+	17 (38.64)	4 (12.90)	13 (100.00)	
Neutropenia	−	30 (68.18)	23 (74.19)	7 (53.85)	0.3334
	+	14 (31.82)	8 (25.81)	6 (46.15)	
Macrocytic anemia	−	29 (65.91)	21 (67.74)	8 (61.54)	0.9621
	+	15 (34.09)	10 (32.26)	5 (38.46)	
Elevated transaminases	−	38 (86.36)	26 (83.87)	12 (92.31)	0.7929
	+	6 (13.64)	5 (16.13)	1 (7.69)	
Mean corpuscular volume	Median	100 [94.0, 102.6]	99.35 [93.70, 102.8]	100 [95.5, 101.7]	0.7812
Blood ammonia (µmol/L)	Median	73.5 [47.75, 273.50]	55.5 [42.00, 90.25]	337.5 [259.50, 563.25]	0.0001

#### 3.3.2 Imaging examinations

Of the 45 patients, 31 underwent cranial ultrasound, CT, or MRI scans (Some parents are uncooperative or complete follow-up at a later stage.). Among them, 27 cases showed imaging findings suggestive of hypoxic-ischemic encephalopathy, subarachnoid hemorrhage, or delayed myelination. Among them, there are 18 cases of combined MMA (18/22) and 9 cases of isolated MMA (9/9). Additionally, 3 patients exhibited significant hydrocephalus. Electroencephalograms (EEGs) were performed in 19 patients, with 15 cases showing epileptiform discharges, spike-and-slow wave complexes, or delayed maturation of brain electrical activity. Both neuroimaging and EEG findings lacked specific diagnostic features.

#### 3.3.3 Blood metabolic screening and urinary organic acid analysis

All 45 patients underwent blood tandem mass spectrometry screening for inherited metabolic disorders, urinary organic acid analysis, and plasma homocysteine measurement. Elevated propionylcarnitine (C3; reference: 0.5–4.7 µmol/L) was observed in 30 patients (66.7%), increased C3/C0 ratio (reference: 0.01–0.20) in 32 cases (71.1%), and elevated C3/C2 ratio (reference: 0.03–0.20) in 38 cases (84.4%). Decreased free carnitine (C0; reference: 7.0–51.4 µmol/L) was found in 11 patients (24.4%). Reduced methionine levels (reference: 8–50 µmol/L) were detected in 11 cases (24.4%), all of whom had combined type MMA. Urinary organic acid analysis revealed elevated levels of 3-hydroxypropionic acid, methylmalonic acid, and methylcitric acid in varying degrees across all 45 patients.

When comparing combined type and isolated type MMA, patients with combined type MMA had significantly lower methionine levels (*p* < 0.05). In contrast, patients with isolated type MMA showed significantly lower C0 levels and higher C3/C0 ratios (both *p* *<* 0.05). No statistically significant differences were found between the two groups in other blood or urine metabolic screening indicators (Table 4).

**Table 4:** Comparison of genetic metabolic screening results between combined type and isolated type MMA groups.

Variable	Level	Total (*n* = 45)	Combined Type (*n* = 32)	Isolated Type (*n* = 13)	*p* value
Methionine	Median	11.74 [6.24, 19.69]	9.27 [5.37, 15.07]	19.21 [14.68, 23.55]	0.0061
C0	Median	10.95 [6.08, 17.70]	14.23 [8.27, 18.38]	4.8 [3.27, 7.97]	0.0014
C3	Mean ± SD	6.83 ± 2.98	7 ± 3.36	6.36 ± 1.56	0.4188
C3/C0	Median	0.78 [0.43, 1.10]	0.55 [0.26, 0.80]	1.38 [0.98, 1.60]	0.0002
C3/C2	Mean	1.09 (0.78)	1.11 (0.87)	1.03 (0.48)	0.7020

### 3.4 Genetic analysis

All 45 patients underwent whole-exome sequencing using next-generation sequencing technology. Among them, 32 cases were identified as combined type MMA. Of these, 31 patients carried pathogenic variants in the *MMACHC *gene and 1 patient had a pathogenic variant in the* ABCD4* gene. A total of 18 distinct* MMACHC* pathogenic variants were detected, including 12 previously reported variants (c.609G>A, c.658_660delAAG, c.567dupT, c.80A>G, c.1A>G, c.481C>T, c.482G>A, c.599G>A, c.666C>A, c.315C>G, c.331C>T, and c.394C>T) and 6 novel variants (c.*2112G>A, c.373G>A, c.656_658del, c.166_174del, c.271dupA, and c.388_390del). The most common variant was c.609G>A, identified in 12 patients (38.7%), of whom 4 were homozygous for this pathogenic variant. In the patient with *ABCD4* pathogenic variant, two known pathogenic variants were detected: c.503C>T and c.998C>T.

Pathogenic variants in the* MMUT* gene were identified in 13 patients, involving a total of 16 distinct variants. Among these, 12 were previously reported pathogenic variants (c.729_730insTT, c.323G>A, c.1084-10A>G, c.1106G>A, c.1233_1235del, c.1677-1G>A, c.1853T>C, c.1741C>T, c.1880A>G, c.626dupC, and c.914T>C), while 4 were novel pathogenic variants (c.178G>A, c.424A>G, c.1091_1108delinsCC, and c.1151C>T). In addition, exon 13 deletion of *MMUT* was detected in 2 patients. The most frequent variant was c.729_730insTT, found in 6 patients (46.2%), including 1 patient who carried a homozygous c.729_730insTT pathogenic variant.

### 3.5 Treatment and outcomes

Among the 45 patients, 5 died during hospitalization, primarily due to respiratory distress and hyperammonemia. An additional 8 patients were discharged against medical advice and subsequently died. Of these 13 deceased patients, 7 (53.8%) had isolated MMA and 6 (18.8%) had combined MMA. Comparison between the two groups revealed a significantly higher proportion of death or discharge against medical advice in patients with isolated MMA, with a statistically significant difference (*P <* 0.05) (Table 1).

## 4 Discussion

Methylmalonic acidemia (MMA) is the most common organic acid metabolism disorder in China in Shandong and Henan province, first reported in 1967 (2). Data from various countries worldwide indicate an estimated MMA incidence ranging from 1:48,000 to 1:250,000 (3).Within China, the incidence exhibits a pattern of being lower in the south and higher in the north (4, 5).

Clinically, MMA can be classified into isolated MMA and combined MMA, based on the presence or absence of hyperhomocysteinemia. Previous studies indicate that the majority of MMA patients in China present with early-onset disease, typically manifesting within the first year of life, and frequently during the neonatal period (6). Combined MMA is the predominant clinical subtype in China, primarily associated with mutations in the “*MMACHC*” gene, while isolated MMA accounts for approximately 30% of cases and is mainly linked to mutations in “*MMUT*” (1, 7, 8). From the perspective of clinical phenotypes, isolated MMA is more prone to recurrent feeding intolerance, vomiting, convulsions and other manifestations than combined MMA, which further predisposes patients to metabolic crises. Long-term sequelae such as intellectual disability and motor developmental retardation are also more common in isolated MMA. Additionally, hyperammonemia is more prominent and refractory in patients with isolated MMA, with persistently and markedly elevated blood ammonia levels that easily trigger acute encephalopathy. In comparison, hyperammonemia in most patients with combined MMA is relatively mild and more amenable to correction. These clinical characteristics were also consistent with the findings of the present study. Isolated MMA presents more typical early warning signs during the neonatal period. Affected newborns tend to develop unexplained poor sucking, recurrent vomiting, tachypnea, hypotonia and progressive drowsiness shortly after birth, accompanied by abnormal laboratory parameters including elevated methylmalonic acid and markedly increased blood ammonia. In contrast, neonatal-onset combined MMA usually manifests with non-specific and milder initial symptoms, making it more likely to be missed in early clinical screening.

The clinical presentation of neonatal-onset MMA is often non-specific, leading to frequent misdiagnosis, missed diagnosis, and delayed confirmation (1, 8). Previous reports indicate that about one-third of MMA patients exhibit non-specific symptoms during the neonatal period, including asphyxia, respiratory distress, feeding difficulties, seizures, and vomiting. These symptoms often lead to misdiagnosis as conditions such as hypoxic-ischemic encephalopathy, respiratory distress syndrome, sepsis, anemia, or gastrointestinal disorders, posing significant diagnostic challenges (9, 10). The clinical phenotypes observed in MMA patients in this study were consistent with previous reports, with feeding difficulties, failure to thrive, jaundice, and respiratory distress being the predominant manifestations. Furthermore, this study found that neonatal-onset MMA primarily occurred in full-term infants. Isolated MMA patients presented at an earlier age and exhibited a significantly higher prevalence of respiratory distress compared to combined MMA patients. This finding aligns with previous reports describing *MMUT* variant patients as having severe, rapidly progressing disease with high early mortality (5, 6, 9, 10).

Accumulation of methylmalonic acid in MMA patients can cause bone marrow suppression, leading to leukopenia, thrombocytopenia, and potentially macrocytic anemia due to folate deficiency. Furthermore, the buildup of methylmalonyl-CoA inhibits hepatic N-acetylglutamate synthase (NAGS) and consumes acetyl-CoA required for N-acetylglutamate synthesis. This inhibition of the urea cycle results in hyperammonemia (11). In this study, isolated MMA patients exhibited significantly lower white blood cell counts and significantly higher plasma ammonia levels compared to combined MMA patients.

In combined MMA, impaired cobalamin cofactor metabolism leads to defective methylcobalamin synthesis. Methylcobalamin is a coenzyme for the conversion of homocysteine to methionine; its deficiency consequently causes hyperhomocysteinemia and reduced methionine levels. This mechanism was corroborated in this study, as methionine levels measured by liquid chromatography-tandem mass spectrometry (LC-MS/MS) were significantly lower in combined MMA patients than in isolated MMA patients. Additionally, isolated MMA patients showed significantly lower free carnitine (C0) levels and significantly higher C3/C0 ratios in their acylcarnitine profiles compared to combined MMA patients. This finding indirectly reflects higher methylmalonic acid levels and greater depletion of free carnitine in isolated MMA, correlating with its more severe clinical phenotype.

In China, isolated MMA accounts for approximately 30% of cases (12). Among the 45 patients in this study, 13 (28.9%) had isolated MMA, consistent with previous reports. Liu et al. identified c.729_730insTT as a hotspot mutation in northern Chinese isolated MMA patients and c.1280G>A as a hotspot in southern Chinese patients (13). This study detected 16 distinct mutations in the 13 patients, including c.729_730insTT in 6 patients, aligning with prior research. Current evidence indicates that the c.729_730insTT mutation is the most common vitamin B12-nonresponsive mutation and is associated with a poor prognosis (7). Combined MMA remains the predominant form in Chinese MMA patients, with *MMACHC* gene mutations being the most frequent. The *MMACHC* c.609G>A mutation is a hotspot among Chinese MMA patients. Yu et al.'s clinical study of 720 MMA patients carrying the c.609G>A mutation confirmed its association with early-onset cblC-type MMA (14). Other common *MMACHC* mutations include c.482G>A, c.658_660delAAG, c.80A>G, and c.217C>T (14). Notably, patients with the c.482G>A mutation often present with milder disease. In this cohort of 31 *MMACHC* patients, 17 (54.8%) carried c.609G>A (4 homozygous), while only 3 patients (6.4% of total cohort) carried c.482G>A. This suggests that c.482G>A is not a common mutation in neonatal-onset MMA, consistent with its association with later-onset forms.

## 5 Conclusions

Neonatal-onset MMA presents with non-specific clinical phenotypes, predominantly affects full-term infants, and is prone to misdiagnosis, leading to delayed treatment and potential multi-organ damage. Therefore, early detection, diagnosis, and intervention are crucial. Newborn screening and urine organic acid analysis are effective methods for early detection. Measurement of homocysteine levels provides significant assistance in identifying combined MMA. Genetic analysis remains the gold standard for definitive diagnosis. For neonates experiencing perinatal asphyxia, unexplained neutropenia, hyperammonemia, seizures, or other atypical manifestations, prompt investigation with plasma amino acid analysis, acylcarnitine profiling, homocysteine measurement, and urine organic acid analysis is essential. Early identification of the underlying cause and initiation of treatment are paramount for improving outcomes.

## Data Availability

The raw data supporting the conclusions of this article will be made available by the authors, on request and without undue reservation.
